# Relationships between cognitive function and body composition among community-dwelling older adults: a cross-sectional study

**DOI:** 10.1186/s12877-017-0651-9

**Published:** 2017-11-02

**Authors:** Hye-Mi Noh, Sohee Oh, Hong Ji Song, Eun Young Lee, Jin-Young Jeong, Ohk-Hyun Ryu, Kyung-Soon Hong, Dong-Hyun Kim

**Affiliations:** 1 Department of Family Medicine, Hallym University Sacred Heart Hospital, College of Medicine, Hallym University, Anyang, South Korea; 2 Department of Biostatistics, Seoul Metropolitan Government Seoul National University Boramae Medical Center, Seoul, South Korea; 3 Hallym Research Institute of Clinical Epidemiology, Hallym University, Chuncheon, South Korea; 4 Department of Endocrinology, Chuncheon Sacred Heart Hospital, College of Medicine, Hallym University, Chuncheon, South Korea; 5 Department of Cardiology, Chuncheon Sacred Heart Hospital, College of Medicine, Hallym University, Chuncheon, South Korea; 6 Department of Social and Preventive Medicine, Hallym University College of Medicine, Chuncheon, South Korea

**Keywords:** Body fat, Cognition, Dual-energy X-ray absorptiometry, Obesity, Older adult

## Abstract

**Background:**

Previous studies reported mixed results regarding the association between cognition and body weight in late life. We evaluated the relationships between cognitive function and body composition among community-dwelling older adults.

**Methods:**

Three hundred twenty subjects (≥65 years, women 53%) with available data of cognitive function and body composition from 2010 Hallym Aging Study. Cognitive function was assessed using Korean Mini-Mental State Examination (K-MMSE). Dual-energy X-ray absorptiometry (DEXA) was used for measuring body composition including body fat and lean body mass. Anthropometric measurements and laboratory data were collected in clinical examination. Body composition variables were divided into sex-specific tertiles, and examined by multivariable logistic regression.

**Results:**

Among female, the highest tertile group of fat mass and second tertile group of total lean body mass were associated with lower risk for cognitive impairment compared to the respective first tertile groups (odds ratios, 0.23 and 0.09, respectively; 95% confidence intervals, 0.04–0.88 and 0.01–0.44, respectively) after adjusting for confounding factors. In male, higher arm bone mineral content was associated with lower risk for cognitive impairment, but significance was lost after adjusting for adiponectin, age, and education.

**Conclusions:**

Higher fat mass and lean body mass were associated with lower risk of cognitive impairment in older women. These observations suggest that body fat and lean mass later in life might be beneficial for cognition.

**Electronic supplementary material:**

The online version of this article (10.1186/s12877-017-0651-9) contains supplementary material, which is available to authorized users.

## Background

Dementia is a major cause of disability and one of the largest disease burdens in the older adult population. As the world’s population is rapidly aging, the number of people affected by dementia worldwide is expected to increase from 30 million in 2010 to 106 million in 2050 [1]. In Korea, the proportion of the population composed of older adults reached 7.2% in 2000, and is predicted to reach 14.3% by 2018, which is the most rapid aging of any nation [2]. Therefore, the burden of dementia in Korea will rapidly increase in the near future. Cognitive decline is a key feature of dementia, and early detection of cognitive impairment allows administration of appropriate treatment and slows the progression of dementia [3, 4]. Identification of risk factors associated with cognitive impairment and early intervention are important issues in the field of primary care for older adults.

Previous studies regarding the association between cognitive performance and body weight status reported inconsistent results. A large, retrospective cohort study in the UK reported that subjects with a higher body mass index (BMI) have a lower risk of dementia, suggesting the “obesity paradox” in dementia [5]. In addition, in an Italian population-based study, a higher BMI and higher body fat mass (BFM) were positively associated with better performance on cognitive tasks related to selective attention and executive functions [6]. The population-based, prospective Mayo Clinic Study of Aging suggested that a greater decrease in BMI per decade was associated with the mild cognitive impairment [7]. In the Sydney Memory and Aging Study, BMI and body fat measured by dual-energy X-ray absorptiometry (DEXA) were related to executive function, suggesting cognitive benefits in overweight older adults [8]. However, a longitudinal study (The TREVISO LONGEVA (TRELONG) Study) among northern Italian adults aged ≥70 years suggested that a high baseline BMI is risk factor for cognitive decline [9]. Few studies have involved Asian populations, which have lower BMI thresholds for overweight and obesity [10]. A Chinese study reported that central obesity or low BMI were significantly associated with the risk of cognitive impairment [11].

Although most previous studies used BMI to diagnose obesity, aging is accompanied by various changes in the body, such as decreasing height, lean body mass, and body fluid, and increasing fat mass [12, 13]. Therefore, BMI might not precisely reflect adiposity in older adults. Given these limitations of BMI, we used DEXA as a more accurate measure of body composition. To our knowledge, this is the first investigation of the relationships between DEXA measurements of body fat and cognitive performance in older Asian adults. Most previous studies did not consider geriatric depression, which might act as a confounding factor for declining cognition and decreasing body weight in the older adult population [14]. We adjusted for a wide range of covariates, including geriatric depression scale (GDS) and metabolic parameters.

In this study, we aimed to determine relationships between body phenotype and composition and cognitive function in older Korean adults, using 2010 data from the Hallym Aging Study.

## Methods

### Study population

The study population was selected from the Hallym Aging Study (HAS), details of which have been published elsewhere [15, 16]. Briefly, HAS is a prospective cohort consisting of community-dwelling adults in a small city in South Korea. The city was divided into 1408 areas based on the 2000 census, and 200 areas were selected randomly for this study. By systematic sampling, 30% of participants were sampled from adults aged 45–64 years, and 70% from adults aged ≥65 years. The first-wave survey in 2003 included 1520 subjects who did not have dementia. Among them, 918 participated in an in-depth clinical study in 2004. Among these 918 subjects, 547 agreed to participate in the 2007 follow-up examinations, and 382 agreed to participate in the 2010 follow-up examinations. We used the 2010 HAS data. The final study population was comprised of 320 subjects (≥65 years, women 53%) with available body composition and anthropometric data. The Institutional Review Board of Hallym University approved the study procedures, and all participants provided informed consent prior to enrollment in the study. The IRB allowed a legally authorized representative to provide informed consent on behalf of a decisionally impaired older adult (Additional file 1).

### Measurements

To assess cognitive function, we used the Korean Mini-Mental State Examination (K-MMSE), which has been widely used in clinical evaluations and research in Korea [17]. K-MMSE consists of 23 items with a possible score of 30 points (5 for time orientation, 5 for place-orientation, 3 for memory registration, 5 for concentration and calculation, 3 for reminiscence, 8 for language and 1 for visual organization). Trained interviewers administered the K-MMSE at a separate space where the subject would not be disturbed for about10 minutes. Cognitive impairment was defined as below the 16th percentile using the age and education criteria [18].

Body fat and lean body mass were quantified by DEXA (Lunar, GE, Fairfield, CT, USA). Lean body mass consisted of skeletal muscle mass and bone mineral content. Anthropometric data were measured for subjects wearing light clothes and no shoes. Height was measured to the nearest 0.1 cm and weight to the nearest 0.1 kg in the upright position. BMI was calculated as weight divided by height squared (kg/m^2^). Waist circumference was measured at the midpoint between the upper end of the iliac crest and the lower end of the 12th rib at the end of each subject’s normal expiration to the nearest 0.1 cm using anthropometric tape. The waist-to-height ratio was calculated as waist circumference (cm)/height (cm). Blood pressure was measured using a standard protocol. All measurements were performed by a trained clinical team and quality control was monitored regularly.

Data regarding age, education level, medical history, and lifestyle factors, including smoking status, alcohol drinking, and regular exercise, were obtained using structured questionnaires administered in a face-to-face manner by trained interviewers. Education level was determined as a continuous variable. Individuals were classified into groups with “no formal education,” “1–5 years of education,” and “≥6 years of education”.

Fasting plasma glucose, total cholesterol (T-Chol), high-density lipoprotein cholesterol (HDL-C), and triglyceride (TG) levels were measured using an auto-analyzer (Hitachi 747, Hitachi, Tokyo, Japan). Low-density-lipoprotein cholesterol (LDL-C) was calculated by the Friedwald eq. (LDL-C = T-Chol–(HDL-C + TG/5)). The homeostasis model assessment of insulin resistance (HOMA-IR) was calculated using the following equation: fasting serum insulin (μU/mL) × fasting plasma glucose (mg/dL)/405. Adiponectin was measured using plasma samples stored at −70 °C by immunoassay (VersaMax, Molecular Devices, Sunnyvale, CA, USA). Glomerular filtration rate (GFR) was estimated using the four-variable Modification of Diet in Renal Disease (MDRD) Study equation.

### Statistical analyses

Data are expressed as means ± standard deviations (SD) for continuous variables and frequencies (%) for categorical variables. The assumption of normality of the data was evaluated by the Shapiro–Wilk test, and a *p-*value greater than 0.05 indicated that the observed distribution of a variable was not significantly different from the normal distribution. HDL-C, TG, insulin, HOMA-IR, adiponectin levels, and K-MMSE were log-transformed, because they did not fit a normal distribution. We used Student’s *t*-test to compare continuous variables and the χ^2^ test or Fisher’s exact test to compare categorical variables between groups. We compared the demographic, metabolic, and body composition characteristics according to the presence of cognitive impairment in each sex. The independent variables that showed statistically significant differences between groups were included in the logistic regression model as covariates. Body composition variables were divided by sex-specific tertiles. Multivariable logistic regression with Firth’s penalized likelihood method was performed to estimate the adjusted odds ratio (OR) and 95% confidence interval (CI), and to assess the association between cognitive impairment and body composition. A *p*-value <0.05 was considered to indicate statistical significance. IBM SPSS Statistics version 20 (IBM Corp., Armonk, NY, USA) and R version 2.15.2 (http://www.r-project.org) were used for statistical analyses.

### Results

The prevalence rates of cognitive impairment were 5.92% in males and 14.29% in females. Body fat percentage and fat mass were higher in females, whereas lean body mass, skeletal muscle mass, and bone mineral content (BMC) were higher in males. There was a significant difference in K-MMSE score, body phenotype and composition, and most of potentially confounding independent variables between males and females (Table 1). Therefore, we analyzed the data according to sex.

**Table 1:** General characteristics of the subjects

	Males (*n* = 152)	Females (*n* = 168)	*p*-value
Age (years)	75.67 ± 5.36	74.44 ± 4.73	0.030
BMI (kg/m^2^)	24.07 ± 2.99	25.26 ± 3.57	0.001
Height (cm)	162.08 ± 6.53	147.74 ± 5.62	<0.001
Waist/height ratio	0.54 ± 0.05	0.58 ± 0.07	<0.001
Body composition			
Body fat (%)	24.89 ± 7.74	34.02 ± 8.52	<0.001
Fat mass (kg)	16.94 ± 4.95	19.46 ± 5.67	<0.001
Total LBM (kg)	43.92 ± 6.01	34.27 ± 4.55	<0.001
Arm LBM (kg)	4.96 ± 0.88	3.61 ± 0.75	<0.001
Leg LBM (kg)	13.83 ± 2.33	10.29 ± 1.70	<0.001
Total ASM (kg)	17.53 ± 2.89	13.06 ± 2.18	<0.001
Arm SM (kg)	4.59 ± 0.86	3.38 ± 0.73	<0.001
Leg SM (kg)	12.93 ± 2.19	9.68 ± 1.59	<0.001
Arm BMC (kg)	0.37 ± 0.27	0.23 ± 0.16	<0.001
Leg BMC (kg)	0.89 ± 0.17	0.61 ± 0.14	<0.001
SBP (mmHg)	137.8 ± 16.12	138.95 ± 16.74	0.535
DBP (mmHg)	78.04 ± 9.05	77.77 ± 8.44	0.781
Fasting glucose (mg/dL)	100.68 ± 25.76	98.82 ± 29.44	0.550
HbA_1_C (%)	5.96 ± 0.91	5.93 ± 0.75	0.798
Fasting insulin (μU/mL)	1.5 ± 0.81	1.77 ± 0.7	0.002
HOMA-IR	0.09 ± 0.9	0.34 ± 0.77	0.009
LDL cholesterol (mg/dL)	111.32 ± 34.62	115.69 ± 32.98	0.251
HDL cholesterol (mg/dL)	3.85 ± 0.26	3.87 ± 0.25	0.411
Triglyceride (mg/dL)	4.79 ± 0.56	4.85 ± 0.53	0.320
Albumin (g/dL)	4.47 ± 0.36	4.47 ± 0.31	0.923
hs-CRP (mg/L)	0.38 ± 1.4	0.22 ± 0.65	0.194
Testosterone (ng/ml)	5.2 ± 1.86	0.12 ± 0.13	<0.001
e-GFR (mL/min/per 1.73 m^2^)	78.54 ± 18.14	85.42 ± 19.18	0.001
Adiponectin (μg/mL)	2.08 ± 0.56	2.34 ± 0.51	<0.001
Hemoglobin (g/dL)	14.39 ± 1.65	13.04 ± 1.08	<0.001
GGT (IU/L)	34.09 ± 34.42	24.19 ± 28.59	0.006
Uric acid (mg/dL)	5.38 ± 1.48	4.23 ± 1.17	<0.001
Comorbidities on treatment			
Hypertension (%)	75 (49.34%)	90 (53.57%)	0.450
DM (%)	26 (17.11%)	22 (13.10%)	0.316
Dyslipidemia (%)	63 (42.28%)	107 (63.69%)	<0.001
CVA (%)	15 (9.87%)	11 (6.55%)	0.278
MI (%)	14 (9.21%)	5 (2.98%)	0.018
Lifestyle			
Smoking (packs/year)	30.27 ± 28.56	0.65 ± 4.23	<0.001
Alcohol (g/week)	152.74 ± 362.32	7.94 ± 33.44	<0.001
Exercise (%)			<0.001
< 3 times/week	105 (69.08%)	152 (90.48%)	
3–4 times/week	14 (9.21%)	7 (4.17%)	
> 4 times/week	33 (21.71%)	9 (5.36%)	
Education			<0.001
0	12 (7.89%)	65 (38.69%)	
1–5 (years)	17 (11.18%)	34 (20.24%)	
≥ 6 (years)	123 (80.92%)	69 (41.07%)	
GDS	13.57 ± 6.9	16.58 ± 6.87	<0.001
K-MMSE	26.6 ± 3.11	23.98 ± 5.3	<0.001
Log (31-K-MMSE)	1.25 ± 0.71	1.63 ± 0.86	<0.001
Cognitive impairment^a^	9 (5.92%)	24 (14.29%)	0.014

*Abbreviations*: *BMC* Bone mineral content, *BMI* Body mass index, *CVA* Cerebrovascular accident, *DBP* diastolic blood pressure, *DM* Diabetes mellitus, *e-GFR* estimated glomerular filtration rate, *GDS* geriatric depression scale, *GGT* gamma glutamyl transferase, *HDL* High-density lipoprotein, *HOMA-IR* homeostasis model assessment of insulin resistance, *hs-CRP* High-sensitivity C-reactive protein, *K-MMSE* Korean Mini-Mental State Examination, *LBM* Lean body mass, *LDL* Low-density lipoprotein, *MI* Myocardial infarction, *SBP* systolic blood pressure, *SM* skeletal muscle mass. All values are means ± SDs or n (%). *p*-values were calculated by Student’s *t*-test or χ^2^ test. Adiponectin, HDL, HOMA-IR, insulin, triglyceride, K-MMSE were log-transformed because they were not normally distributed. The data describing comorbidities and treatments were obtained from structured questionnaires. ^a^Cognitive impairment was defined by lower than the 16th percentile using the age and education criteria

The sex-specific general characteristics of the study subjects according to the presence of cognitive impairment are presented in Table 2. Mean age was significantly higher in the cognitive impairment group compared to the normal cognition group in both sexes. Among body phenotypes, BMI was higher only in female with normal cognition than in the cognitive impairment group (25.52 ± 3.58 and 23.73 ± 3.14, respectively, *p = 0.016)*, whereas waist/height ratio was not significantly different between groups. Height was greater in the normal cognition group in both sexes.

**Table 2:** Characteristics of the study subjects with or without cognitive impairment according to sex

	Males			Females		
	Cognitive impairment			Cognitive impairment		
	Yes (*n* = 9)	No (*n* = 143)	*p*-value	Yes (*n* = 24)	No (*n* = 144)	*p*-value
Age (year)	80.11 ± 7.79	75.39 ± 5.09	0.038	76.29 ± 4.59	74.13 ± 4.7	0.033
BMI (kg/m^2^)	22.37 ± 3.87	24.18 ± 2.91	0.109	23.73 ± 3.14	25.52 ± 3.58	0.016
Height (cm)	157.97 ± 5.99	162.34 ± 6.49	0.048	145.88 ± 5.31	148.05 ± 5.63	0.049
Waist/height ratio	0.51 ± 0.07	0.54 ± 0.05	0.203	0.57 ± 0.06	0.58 ± 0.07	0.390
Body composition						
Body fat (%)	20.12 ± 7.16	25.19 ± 7.7	0.076	31.38 ± 9.06	34.46 ± 8.38	0.076
Fat mass (kg)	14.03 ± 5.48	17.12 ± 4.88	0.082	16.97 ± 4.96	19.87 ± 5.69	0.014
Total LBM (kg)	41.98 ± 7.15	44.05 ± 5.94	0.480	33.31 ± 5.59	34.43 ± 4.36	0.029
Arm LBM (kg)	4.54 ± 0.98	4.99 ± 0.87	0.243	3.54 ± 0.82	3.63 ± 0.74	0.362
Leg LBM (kg)	13.22 ± 3.00	13.87 ± 2.29	0.637	10.09 ± 1.95	10.33 ± 1.66	0.180
Total ASM (kg)	16.65 ± 3.66	17.58 ± 2.84	0.615	12.80 ± 2.51	13.11 ± 2.13	0.255
Arm SM (kg)	4.25 ± 0.92	4.61 ± 0.85	0.305	3.33 ± 0.76	3.39 ± 0.72	0.367
Leg SM (kg)	12.40 ± 2.83	12.97 ± 2.15	0.665	9.48 ± 1.80	9.72 ± 1.56	0.180
Arm BMC (kg)	0.29 ± 0.07	0.37 ± 0.28	0.018	0.21 ± 0.07	0.23 ± 0.17	0.456
Leg BMC (kg)	0.82 ± 0.21	0.90 ± 0.16	0.195	0.61 ± 0.17	0.61 ± 0.14	0.472
SBP (mmHg)	138.67 ± 20.55	137.75 ± 15.89	0.509	140.62 ± 19.8	138.67 ± 16.24	0.702
DBP (mmHg)	75.67 ± 12.1	78.19 ± 8.86	0.392	76.92 ± 10.99	77.91 ± 7.98	0.908
Fasting glucose (mg/dL)	112.89 ± 59.47	99.9 ± 22.22	0.745	94.42 ± 16.76	99.55 ± 31.04	0.605
HbA_1_C (%)	5.58 ± 0.27	5.98 ± 0.93	0.195	5.77 ± 0.58	5.96 ± 0.77	0.147
Fasting insulin (μU/mL)	1.22 ± 1.13	1.52 ± 0.78	0.205	1.71 ± 0.65	1.78 ± 0.71	0.295
HOMA-IR	−0.13 ± 1.31	0.1 ± 0.87	0.407	0.24 ± 0.63	0.35 ± 0.79	0.338
LDL cholesterol (mg/dL)	102.11 ± 19.92	111.91 ± 35.32	0.372	114.12 ± 38.78	115.95 ± 32.06	0.924
HDL cholesterol (mg/dL)	3.94 ± 0.24	3.84 ± 0.26	0.232	3.88 ± 0.32	3.87 ± 0.24	0.861
Triglyceride (mg/dL)	4.66 ± 0.54	4.8 ± 0.57	0.491	4.71 ± 0.67	4.87 ± 0.51	0.117
Albumin (g/dL)	4.43 ± 0.41	4.48 ± 0.36	0.606	4.33 ± 0.41	4.49 ± 0.28	0.051
hs-CRP (mg/L)	0.2 ± 0.16	0.4 ± 1.44	0.738	0.62 ± 1.62	0.16 ± 0.19	0.138
Testosterone (ng/ml)	4.79 ± 2.33	5.23 ± 1.83	0.582	0.11 ± 0.1	0.12 ± 0.13	0.903
e-GFR (mL/min/per 1.73 m^2)^	88.7 ± 19.58	77.88 ± 17.92	0.057	76.16 ± 23.62	86.96 ± 17.98	0.017
Adiponectin (μg/mL)	2.48 ± 0.46	2.05 ± 0.55	0.026	2.37 ± 0.51	2.33 ± 0.51	0.724
Hemoglobin (g/dL)	14.31 ± 1.5	14.4 ± 1.66	0.776	12.66 ± 1.41	13.1 ± 1.01	0.236
GGT (IU/L)	50 ± 70.17	33.07 ± 30.99	0.304	30.21 ± 40.3	23.19 ± 26.19	0.678
Uric acid (mg/dL)	4.84 ± 1.2	5.41 ± 1.49	0.275	4.29 ± 1.19	4.22 ± 1.17	0.879
Comorbidities on treatment						
Hypertension (%)	3 (33.33%)	72 (50.35%)	0.495	8 (33.33%)	82 (56.94%)	0.032
DM (%)	2 (22.22%)	24 (16.78%)	0.652	2 (8.33%)	20 (13.89%)	0.744
Dyslipidemia (%)	2 (22.22%)	61 (43.57%)	0.303	16 (66.67%)	91 (63.19%)	0.743
CVA (%)	1 (11.11%)	14 (9.79%)	1.000	2 (8.33%)	9 (6.25%)	0.659
MI (%)	0 (0.00%)	14 (9.79%)	1.000	4 (16.67%)	1 (0.69%)	0.001
Lifestyle						
Smoking (pack/year)	32.41 ± 39.12	30.14 ± 27.94	0.842	1.12 ± 5.47	0.57 ± 4	0.849
Alcohol (g/week)	148.21 ± 238.27	153.03 ± 369.32	0.780	2.35 ± 8.68	8.87 ± 35.88	0.415
Exercise			0.722			0.705
< 3 times/week	7 (77.78%)	98 (68.53%)		21 (87.50%)	131 (90.97%)	
≥ 3 times/week	2 (22.22%)	45 (31.47%)		3 (12.50%)	13 (9.03%)	
Education			1.000			0.002
< 6 (years)	1 (11.11%)	28 (19.58%)		21 (87.50%)	78 (54.16%)	
≥ 6 (years)	8 (88.89%)	115 (80.42%)		3 (12.50%)	66 (45.83%)	
GDS	15.44 ± 6.69	13.45 ± 6.91	0.394	19.38 ± 4.98	16.11 ± 7.04	0.040

*Abbreviations*: *BMC* Bone mineral content, *BMI* Body mass index, *CVA* Cerebrovascular accident, *DBP* Diastolic blood pressure, *DM* Diabetes mellitus, *e-GFR* Estimated glomerular filtration rate, *GDS* Geriatric depression scale, *GGT* Gamma glutamyl transferase, *HDL* High-density lipoprotein, *HOMA-IR* Homeostasis model assessment of insulin resistance, *hs-CRP* High-sensitivity C-reactive protein, *K-MMSE* Korean Mini-Mental State Examination, *LBM* Lean body mass, *LDL* Low-density lipoprotein, *MI* myocardial infarction, *SBP* Systolic blood pressure, *SM* Skeletal muscle mass. Adiponectin, HDL, HOMA-IR, insulin, and triglyceride were log-transformed because they were not normally distributed. The data describing comorbidities and treatments were obtained from structured questionnaires

With regard to body composition, arm BMC was higher in males in the normal cognition group than in those in the cognitive impairment group (0.37 ± 0.28 and 0.29 ± 0.07, respectively, *p = 0.018*). Fat mass and total lean body mass were significantly higher in females with normal cognitive function than in those in the cognitive impairment group (19.87 ± 5.69 and 16.97 ± 4.96, respectively, *p = 0.014,* 34.43 ± 4.36 and 33.31 ± 5.59, respectively, *p = 0.029)*. In males, adiponectin level was lower in the normal cognition group than the cognitive impairment group (2.05 ± 0.55 and 2.48 ± 0.46, respectively, *p = 0.026*). In females, GDS score and rate of past history of MI were higher in the cognitive impairment group, whereas the prevalence of hypertension and educational level were higher in the normal cognition group.

We divided the body composition variables into sex-specific tertiles, and multivariable logistic regression analysis was performed to estimate the association between cognitive impairment and body composition. Among males, the highest tertile of arm BMC was associated with lower risk for cognitive impairment compared to the first tertile in model 1 (OR 0.10; 95% CI, 0.001–0.88), but significance was lost after adjusting for adiponectin, age, and education (Table 3). In females, the highest tertile group of fat mass and second tertile group of total lean body mass were associated with lower risk for cognitive impairment compared to the respective first tertile groups (OR 0.23 and 0.09, respectively; 95% CI, 0.04–0.88 and 0.01–0.44, respectively) after adjusting for confounding factors (Table 4). A past history of MI was associated with a greater risk of cognitive impairment, whereas treatment of hypertension was associated with lower risk of cognitive impairment. Height was not significantly associated with cognitive impairment in either sex.

**Table 3:** Multivariable adjusted odds ratios of cognitive impairment and body composition among males

	Males		
Parameter	Model 1	Model 2	Model 3
Height	0.93 (0.82, 1.04)	0.95 (0.84, 1.05)	0.95 (0.84, 1.06)
Arm BMC (tertile)			
2nd tertile vs. 1st tertile	0.56 (0.13, 2.12)	0.65 (0.15, 2.54)	0.57 (0.12, 2.24)
3rd tertile vs. 1st tertile	0.10 (0.001, 0.88)	0.12 (0.001, 1.13)	0.14 (0.001, 1.39)
Adiponectin		2.98 (0.82, 12.59)	2.65 (0.70, 11.41)
Age			1.10 (0.97, 1.25)
Education			1.85 (0.35, 19.05)

*Abbreviations*: *BMC* Bone mineral content. Model 1 includes height and arm BMC. Model 2 includes the Model 1 variables plus adiponection. Model 3 includes the Model 2 variables plus age and education

Presented values were odds ratio (95% CI)

**Table 4:** Multivariable adjusted odds ratios of cognitive impairment and body composition among females

	Females		
Parameter	Model 1	Model 2	Model 3
Height	0.97 (0.89, 1.06)	0.99 (0.89, 1.10)	1.03 (0.91, 1.16)
Fat mass (tertile)			
2nd tertile vs. 1st tertile	0.76 (0.25, 2.19)	1.16 (0.35, 3.92)	1.15 (0.33, 4.03)
3rd tertile vs. 1st tertile	0.25 (0.06, 0.83)	0.20 (0.04, 0.80)	0.23 (0.04, 0.88)
Total LBM (tertile)			
2nd tertile vs. 1st tertile	0.28 (0.08, 0.89)	0.10 (0.01, 0.45)	0.09 (0.01, 0.44)
3rd tertile vs. 1st tertile	0.47 (0.14, 1.49)	0.48 (0.13, 1.69)	0.42 (0.11, 1.54)
e-GFR		0.98 (0.95, 1.002)	0.98 (0.95, 1.01)
GDS		1.06 (0.98, 1.17)	1.05 (0.95, 1.16)
HTN		0.34 (0.11, 0.99)	0.31 (0.09, 0.91)
MI		80.89 (7.56, 1910.93)	63.90 (5.42, 1642.46)
Age			1.05 (0.93, 1.19)
Education			0.34 (0.08, 1.20)

*Abbreviations*: *LBM* Lean body mass, *MI* Myocardial infarction, *GDS* Geriatric depression scale. Model 1 includes height, fat mass, and total LBM. Model 2 includes the Model 1 variables plus e-GFR, GDS, HTN, MI. Model 3 includes the Model 2 variables plus age and education

Presented values were odds ratio (95% CI)

## Discussion

In this study, we investigated the relationship between cognitive function and body phenotype and composition. There have been several reports that height is related to cognitive function. An Israeli study reported that shorter stature was associated with lower cognition in elderly men with type 2 diabetes mellitus [19]. Shorter adults tend to have a smaller head circumference, which is associated with poorer brain reserve [20], and brain reserves allow individuals to preserve normal cognitive function despite brain degeneration due to aging [21]. However, in the present study, height was not associated with cognitive impairment in either sex, which was not consistent with the report of Quan et al. using the 2004 HAS data; they reported that the risk of cognitive impairment was fourfold higher in the shortest quartile than in the tallest quartile of men, but not in women [15]. However, this previous report had limitations in that it did not consider body weight or composition as well as various confounding factors, such as comorbidities and laboratory parameters.

We showed that BMI was significantly higher in females with normal cognition group than in the cognitive impairment group. The mean BMI of females with normal cognition was 25.5 kg/m^2^, which was similar to the obesity thresholds used in Asian population [10]. However, waist/height ratio as an indicator of central obesity showed no differences according to cognitive function in either sex. A retrospective cohort study in the UK reported that subjects with a higher BMI had a lower dementia risk [5]. Compared with those of a healthy weight, underweight persons (BMI <20 kg/m^2^) had a 34% higher (95% CI 29–38) risk of dementia. Furthermore, the incidence of dementia decreased for each increase in BMI category, with very obese individuals (BMI >40 kg/m^2^) having a 29% lower (95% CI 22–36) risk of dementia than those of a healthy weight. An Italian population-based study also reported that MMSE was positively associated with BMI and BFM calculated from skin fold thickness in both men and women [6]. Recent systematic reviews suggested an inverse association between obesity defined by BMI in late-life and dementia, consistent with the concept of the obesity paradox. However, they included 21 studies (only one Asian study), which had relatively short follow-up period, and did not consider sex-related differences [22].

Aging is accompanied by various changes in the body—such as decreasing height, lean body mass, and body fluid—whereas fat mass increases; therefore, the BMI in older adults might not precisely reflect their adiposity [12, 13]. Given these limitations of BMI, we used DEXA as a more accurate measure of body composition, and demonstrated that higher fat mass and lean mass were associated with lower risk of cognitive impairment in females.

Several possible mechanisms may explain how obesity, defined by excess adipose tissue, is related to cognitive function. First, body fat contains sex hormones and leptin, which may contribute to prevention of cognitive decline in older adults [23]. Second, a previous study reported that BMI was positively associated with greater white matter volume, suggesting that obesity increases myelin [24]. However, these positive associations between obesity and cognition could also result from the ‘survival bias’—obese individuals who live through middle age may have genes associated with higher cognitive function [25]. In addition, underweight and cognitive impairment might share an etiology. First, dysregulation of hormone secretion has been observed in anorexia, which could lead to a decline in cognitive function [26]. Second, an underweight status in the older adults may indicate preclinical dementia [27]. Third, underweight could also be a result of mental illness, such as depression. Geriatric depression might lead to both a decline in cognition and a decrease in body weight, which could act as confounding factors between cognition and body weight in older adults [14, 28]. However, in our study, the association between cognitive function and fat mass remained significant after adjusting for depression scores.

Previous studies suggested that loss of muscle mass and function, sarcopenia, might be related to brain atrophy and low cognitive performance [29]. We found that the second tertile group of total lean body mass in females showed lower risk for cognitive impairment compared to the first tertile group, however, there was no significant association in the third tertile group. In the Epidemiologie de l’Osteoporose (EPIDOS) cohort study, six definitions of sarcopenia were used, none of which was associated with cognitive impairment. However, a low gait speed and low handgrip strength were associated with cognitive impairment [30].

In the present study, interestingly, we found that a past history of MI was associated with higher risk of cognitive impairment in females, but not in males. However, there was no subject with a history of MI among nine males with cognitive impairment, and it was, therefore, difficult to judge the association between MI and cognitive function. This may be due to the relatively small sample size in our study or survival bias caused by high mortality of MI. Our findings suggested that physicians should pay attention to cognitive decline and consider dementia screening among older adults with a history of MI. The Women’s Health Initiative Memory Study reported that cardiovascular diseases, especially MI or angina, were associated with cognitive decline in elderly postmenopausal women [31]. When MI occurs, the blood supply to the heart and brain is decreased, which leads to loss of grey matter volume and cognitive impairment [32]. Further studies are warranted to investigate the mechanism underlying the association between MI and cognitive impairment.

This study had several limitations. First, it was of a cross-sectional design, which prevented making causal inferences between body composition and cognitive function. Second, because of the relatively small sample size, our results could not well represent the general Korean older adult population. To determine the influence of body composition on cognitive impairment, further large prospective studies are needed. Third, we used the MMSE to assess cognitive function. Although MMSE is the most commonly used screening test for dementia, the results of MMSE may not always match those of a detailed battery of cognitive tests.

Despite these limitations, our study confirmed the associations between specific components of body composition and cognitive function in an older adult Korean population. In addition, we used DEXA as a more accurate measure of body composition, and adjusted for a wide range of confounding factors, such as metabolic parameters and depression.

## Conclusions

We demonstrated that higher fat mass and lean mass were associated with lower risk of cognitive impairment in older adult Korean women. Future studies should clarify the relationship between fat mass at older ages and cognitive function.

## Additional file

Additional file 1: Figure S1. Flowchart of the selection of the study population. (DOCX 23 kb)

## Abbreviations

BMI: Body mass index
DEXA: Dual-energy X-ray absorptiometry
GDS: Geriatric depression scale
HAS: Hallym Aging Study
K-MMSE: Korean Mini-Mental State Examination
