# Supplementary material for: Relationships between cognitive function and body composition among community-dwelling older adults: a cross-sectional study
Source: BMC Geriatr. 2017 Nov 2;17:259. doi: 10.1186/s12877-017-0651-9 (PMC5667483; doi:10.1186/s12877-017-0651-9)
Supplement: Additional file 1: Figure S1. — Flowchart of the selection of the study population. (DOCX 23 kb) [file 12877_2017_651_MOESM1_ESM.docx]

Figure1. Flowchart of the selection of the study population

1,520 subjects (≥45 years) without dementia were included in first-wave survey (2003)

918 subjects in the in-depth clinical study (2004)

547 subjects in the follow-up examinations (2007)

382 subjects in the follow-up examinations (2010)

Excluded

Subjects aged 45–64 years (*n*=62)

320 subjects (≥65 years old)
